# Food By-Products to Extend Shelf Life: The Case of Cod Sticks Breaded with Dried Olive Paste

**DOI:** 10.3390/foods9121902

**Published:** 2020-12-19

**Authors:** Olimpia Panza, Valentina Lacivita, Carmen Palermo, Amalia Conte, Matteo Alessandro Del Nobile

**Affiliations:** Department of Agricultural Sciences, Food and Environment, University of Foggia, via Napoli, 25, 71122 Foggia, Italy; olimpia.panza@unifg.it (O.P.); valentina.lacivita@unifg.it (V.L.); carmen.palermo@unifg.it (C.P.); matteo.delnobile@unifg.it (M.A.D.N.)

**Keywords:** olive oil by-products, breaded fish, fish shelf life, fish quality, sustainable food

## Abstract

Recently, the interest in recovery bioactive compounds from food industrial by-products is growing abundantly. Olive oil by-products are a source of valuable bioactive compounds with antioxidant and antimicrobial properties. One of the most interesting by-products of olive oil obtained by a two-phase decanter is the olive paste, a wet homogeneous pulp free from residuals of the kernel. To valorize the olive paste, ready-to-cook cod sticks breaded with dried olive oil by-products were developed. Shelf-life tests were carried out on breaded cod sticks and during 15 days of storage at 4 °C pH evolution, microbiological aspects, and sensory properties were also monitored. In addition, the chemical quality of both control and active samples was assessed in terms of total phenols, flavonoids, and antioxidant activity. The enrichment with olive paste increased the total phenols, the flavonoids, and the antioxidant activity of the breaded fish samples compared to the control. Furthermore, the bioactive compounds acted as antimicrobial agents, without compromising the sensory parameters. Therefore, the new products recorded a longer shelf life (12 days) than the control fish sample that remained acceptable for nine days.

## 1. Introduction

Seafood is an important part of a healthy diet, being an excellent source of high biological value proteins, omega-3 polyunsaturated acids, vitamins, and mineral salts [1]. Among the various fishery products, salted cod is appreciated by consumers for its taste and nutritional value [2]. However, due to its high concentration of salt, accounting for about 20%, the cod must be rehydrated for a few days and used quickly because the conditions for bacterial growth are favorable due to the high water content and low salt concentration in the product. The water content can be further reduced by drying and when it falls below 50%, so-called dried salted cod is obtained [3]. While it may be true that the salt content and moisture content of the product can reduce the risk of microbial contamination, on the other hand, the desalination process, through the soaking process, can promote microbial and fungal proliferation. Specifically, the chilled soaking process encourages halotolerant psychrotrophs that have survived salting and drying. Predominant microorganisms from soaked cod at refrigeration temperatures include Enterobacteriaceae, *Pseudomonas fluorescens* and *putida*, *Aeromonas hydrophila*, and *Shewanella putrefaciens* [4].

In addition, its time-consuming preparation contrasts with the trend of the modern consumer, who is looking for ready-to-eat fish products or ready-to-cook products. In this context, the idea of developing new cod fillets could gain consensus among consumers [5,6].

Among the preservation methods reported in the abundant literature dealing with seafood shelf life [7,8,9,10], modified atmosphere packaging, chemical preservatives, and non-thermal technologies have been applied to desalted cod [11,12,13].

However, modern consumers are increasingly looking for sustainable, safe, and healthy products [14]. Therefore, fresh and minimally processed food, more natural, produced with the minimum amounts of additives, microbiologically safe and nutritious but at the same time, sustainable foods are highly preferred [15].

In the context of food sustainability, several pieces of research have been conducted with the aim to valorize industrial by-products. In fact, natural preservatives from fruit and vegetable by-products could be valid agents to guarantee the shelf-life extension, since they are rich in bioactive compounds with antimicrobial and antioxidant properties [16,17,18,19]. Among food industrial by-products, there is increasing attention for the application of olive by-products, and in particular semi-solid pomace, being very rich in phenolic compounds with well-known bioactive properties [20,21,22,23,24]. The rising interest in recovering the bioactive compounds of olive-oil industrial by-products for food applications certainly has a dual objective, on the one hand, it allows to adequately manage industrial waste to limit the environmental impact and, on the other hand, it can valorize the beneficial properties and biological activities of polyphenols that are commercially available at very low costs [25,26].

The purpose of our study was to develop breaded ready-to-cook sticks of cod, implementing new and effective combinations between fish and olive-oil by-products. To the aim, the breading was done with and without the olive paste, properly distributed, and then applied on the fish surface. During refrigerated storage, a shelf-life test was carried out, monitoring the quality parameters related to pH evolution, microbiological aspects, and sensory properties to verify if the active breading was able to extend the shelf life of the product. For completeness, the chemical quality of the samples was also assessed, to highlight the difference in terms of phenolic compounds, flavonoids, and antioxidant activity between the control and active products.

## 2. Materials and Methods

### 2.1. Raw Materials

Refrigerated salted cod fillets were purchased from a local market (Manfredonia, FG, Italy). The olive paste from Cellina di Nardò cultivar was obtained at the beginning of the year 2020, in January, from a local olive mill (Lecce, BA, Italy) using a Pieralisi Leopard with Multi-Phase Decanter, which combines modern extraction technology without water addition and recovers a certain quantity of by-product paste, named olive paste, made up of wet pulp without any traces of kernel. The olive paste was immediately dried at 35 °C in a dryer (SG600, Namad, Rome, Italy) for 72 h. The dried olive paste was reduced to a fine powder (<500 μm) by a hammer mill (16/BV-Beccaria s.r.l., Cuneo, Italy) and then stored at 4 °C until its utilization in February 2020. All the ingredients to prepare the cod stick breaded fish, such as the breading, the spices, the potato flakes, and the fresh milk were purchased at a local market (Foggia, Italy).

### 2.2. Breaded Cod Sticks Preparation

Cod fillets were coarsely desalted, soaked, and stored at 4 ± 1 °C for five days, changing the water every day. On the sixth day, cod fillets were drained to eliminate excess water for about half an hour and the skin was removed. Then, the fillets (about 67% *w*/*w* water content and about 1% *w*/*w* NaCl) were cut in sticks of approximately 12 g. Two different mixtures were prepared: (i) the control mixture contained 180 g of breading with fish spices, 180 g of potato flakes; (ii) the active mixture prepared with 180 g of breading with fish spices, 180 g of potato flakes, 90 g of olive paste. The amount of olive paste represents the best optimization with other ingredients to give a final product acceptable after cooking. Control samples (Ctrl) were obtained as follows: after dipping in a solution of water and milk (1:1), samples were breaded in the control mixture, by repeating the passage twice, then were manually compacted, placed above a food tray with a pad and packaged in air using high-barrier film bags (multi-layer film Nylon/Polyethylene) with a thickness of 150 µm, provided by Biochemia (Bari, Italy) and kept under refrigeration (4 ± 1 °C). Active samples (Active) were prepared using the same procedure as for Ctrl samples, using the active mixture. Two sticks were packaged in each bag. A total of 64 sticks were prepared from two different batches: 32 Ctrl samples and 32 Active samples. All samples were stored at 4 ± 1 °C for 15 days.

### 2.3. Chemicals

Folin–Ciocalteu reagent, gallic acid monohydrate, ethanol, ABTS (2,2-azino-bis (3-ethylbenzothiazoline-6-sulfonic acid) diammonium salt), potassium persulfate (K_2_S_2_O_8_), Trolox (6-hydroxy-2,5,7,8-tetramethylchroman-2-carboxylic acid), aluminum chloride (AlCl_3_), sodium nitrite (NaNO_2_), sodium hydroxide solution (NaOH), quercetin, were supplied from Sigma-Aldrich (Milan, Italy). Anhydrous sodium carbonate (Na_2_CO_3_) was supplied from Carlo Erba (Milan, Italy). For the preparation of the phosphate-buffered saline (PBS), the following salts were used: sodium phosphate dibasic heptahydrate (Na_2_HPO_4_·7H_2_O) and sodium phosphate monobasic monohydrate (NaH_2_PO_4_·H_2_O). All reagents were of analytical grade.

### 2.4. Extraction of Bioactive Compounds

For chemical analyses, both raw (R-Ctrl and R-Active) and cooked samples (C-Ctrl and C-Active) (200 °C for 15 min in an electric oven, Europa Forni, Vicenza, Italy), were firstly subjected to drying (35 °C in a ventilated stove, BINDER GmbH, Tuttlingen, Germany), milled to obtain a powder, and then subjected to extraction as reported by Cedola et al. [20].

### 2.5. Determination of Total Phenols Content, Total Flavonoids, and Antioxidant Activity

The evaluations of total phenols and total flavonoids were carried out on both raw and cooked breaded cod sticks. All the chemical analyses were performed the day after the sample preparation. Total phenol content was determined according to the Folin–Ciocalteu method as reported by Cedola et al. [20]. The colorimetric method allowed to quantify the total phenol content as milligrams of gallic acid equivalents (GAE) per gram of dry weight (dw), according to a calibration curve (3.12–100 mg/L; R^2^ = 0.9997).

Total flavonoid content was determined using the aluminum chloride colorimetric method, according to [20]. The measure was carried out at 415 nm with a spectrophotometer (UV1800; Shimadzu Italia s.r.l; Milan, Italy) and the total flavonoids were expressed as milligrams of quercetin equivalent (QE) per gram of dry weight (dw). Quercetin standard solutions were used for constructing the calibration curve (12.5–400 mg/L; R^2^ = 0.9955).

The antioxidant activity of breaded cod sticks was assessed using two methods: ABTS (2,2-azino-bis (3-ethylbenzothiazoline-6-sulfonic acid diammonium salt) assay. The test is based on the ability of antioxidants to interact with the radical cation 2,2’-azino-bis (3-ethylbenzothiazoline-6-sulfonic acid) (ABTS·+) inhibiting its absorption at 734 nm [27], and the analysis was carried out according to the methods used by Cedola et al. [20]. A calibration curve was built using Trolox as the standard, at concentrations between 6.25 mg/L and 500 mg/L (R^2^ = 0.9977) and the antioxidant activity was expressed as milligrams of Trolox equivalents for gram of dry weight (dw). All tests were carried out in triplicate.

### 2.6. Microbiological Analyses and pH Determination

Ctrl and Active samples (20 g) were aseptically weighed into a sterile stomacher bag, diluted with peptone water (dilution 1:10), and homogenized for 90 s with a Stomacher LAB Blender 400 (Pbi International, Milan, Italy). Serial dilutions were plated onto specific media in Petri dishes to enumerate *Pseudomonas* spp., hydrogen sulfide-producing bacteria (HSPB), psychrotolerant and heat-labile aerobic bacteria (PHAB), mesophilic and psychrotrophic bacteria, Enterobacteriaceae and lactic acid bacteria (LAB) according to Danza et al. [28]. The conditions used for counting HSPB and PHAB were suggested by the Nordic Committee on Food Analyses [29]. All media and supplements were obtained from Oxoid (Milan, Italy). The microbiological analyses were carried out twice on two different samples and the results are expressed as Log cfu/g. Microbial thresholds were set to 5 × 10^6^ cfu/g for total viable mesophilic and psychrotrophic bacteria, 10^6^ cfu/g for *Pseudomonas* spp. and *Shewanella*, 10^7^ cfu/g for *Photobacterium* [30]. The fitting of experimental data allowed to quantify the microbiological acceptability limit (MAL), to be intended as the time (day) to reach the specific microbiological threshold. It was calculated according to what was reported by Del Nobile et al. [31].

The measurement of pH was performed in triplicate on the first homogenized dilution of fish samples, using a pH meter (Crison, Barcelona, Spain). Two different samples were used for each measurement. Microbiological analyses and pH were analyzed at the initial time and after 2, 4, 8, 12, and 15 days of refrigerated storage at 4 °C.

### 2.7. Sensory Analysis

The quantitative descriptive analysis (QDA) was used for a sample comparison, according to the guidelines of the Codex Alimentarius Commission. To the aim, breaded cod sticks were submitted to a panel of five trained judges. The panelists have familiar eating habits with fish and fish products and already had experience in the evaluation of burgers and fillets based on fish. They were retrained for two days (2 h session), to establish the appropriate attributes for sensory evaluation and in order to minimize individual differences and ensure repeatability of results. The panelists were asked to give judgments on odor, color, appearance, texture, and overall quality using a nine-point scale. In the scale, 9 corresponded to excellent, 8 to very good, 7 to good, 6 to reasonable, 5 to not good (acceptable limit), 4 to disliked, 3 to bad, 2 to very bad, and 1 to completely unacceptable [32]. Before the sensory analysis, samples were sliced with a knife without removing the breading crust. Samples were differently coded and presented to each panelist simultaneously in random order. The fitting of the experimental data related to the overall quality allowed to quantify the sensory acceptability limit (SAL), which represents the time (day) necessary to reach the sensory threshold. It was calculated according to Del Nobile et al. [31].

### 2.8. Statistical Analysis

Fitting of experimental data provided us MAL and SAL parameters. The lowest value among the MAL and SAL parameters gave us the product shelf life. The experimental data were compared by one-way analysis of variance (ANOVA). A Duncan’s multiple range test, with the option of homogeneous groups (*p* < 0.05), was carried out to determine significant differences among samples. STATISTICA 7.1 for Windows (StatSoft, Inc, Tulsa, OK, USA) was used.

## 3. Results and Discussion

### 3.1. Total Phenols, Total Flavonoids, and Antioxidant Activity of Breaded Cod Sticks

The chemical quality of cod sticks breaded with dry olive paste was assessed in terms of total phenol content (mg GAE/g dw), flavonoids (mg QE/g dw), and antioxidant activity (mg Trolox equivalent/g dw) as shown in Table 1. As can be seen, both raw (R-Ctrl and R-Active) and cooked (C-Ctrl and C-Active) products were analyzed. According to the recorded data, the dry olive paste was able to improve the chemical quality of breaded fish, as total phenols and flavonoids increased in both raw and cooked Active samples. In particular, the total phenol content expressed as Gallic acid equivalents (GAEs) was approximately six times higher in both raw and cooked Active samples (12.63 and 12.46 mg GAE/g dw, respectively) compared to R-Ctrl and C-Ctrl (2.70 and 2.82 mg GAE/g dw, respectively). Moreover, flavonoids are one of the major groups of phenolic compounds present in olive oil by-products [24,33,34]. This trend was also found in our study, in fact, the Active breaded cod sticks showed higher flavonoid content compared to the Ctrl and their concentration varied from 1.69 mg QE/g dw (R-Ctrl) to 13.68 mg QE/g dw (R-Active). Similar results were reported by Cedola et al. [20], who observed that phenols (31.16 mg GAE/g dw) and flavonoids (61.24 mg QE/g dw) recorded in the dry olive paste significantly enriched fish burgers prepared with olive paste in the formulation. As can be seen from Table 1, the flavonoid content in the C-Active samples (10.61 mg QE/g dw) is lower than in R-Active. Probably, the cooking process at 200 °C for 15 min in an electric oven could have destroyed some flavonoids, while keeping them in a higher concentration than the cooked control (1.38 mg QE/g dw). As reported by several authors, the cooking process can affect polyphenols in different ways [35,36]. In some cases, their availability may increase [20,37], in other cases cooking may reduce their content [38]. As a consequence of the enrichment of breading with dry olive paste, an antioxidant capacity was found in the Active breaded cod sticks compared to the Ctrl (20.02 against 5.84 mg Trolox/g dw). Results obtained in our study agree with data reported by Cedola et al. [19], thus confirming that the high concentration of phenols and flavonoids gives fish products a higher antioxidant capacity compared to the control ones. As can be seen, a positive relationship between phenol content and antioxidant activity was also found in our data. This aspect has been confirmed by other authors [22,23,39], who also suggested that the antioxidant capacity of the olive mill waste is related to the content of phenols and to the nature of the phenolic extracts.

### 3.2. Microbial Quality of Breaded Cod Sticks

**Figure 1:**
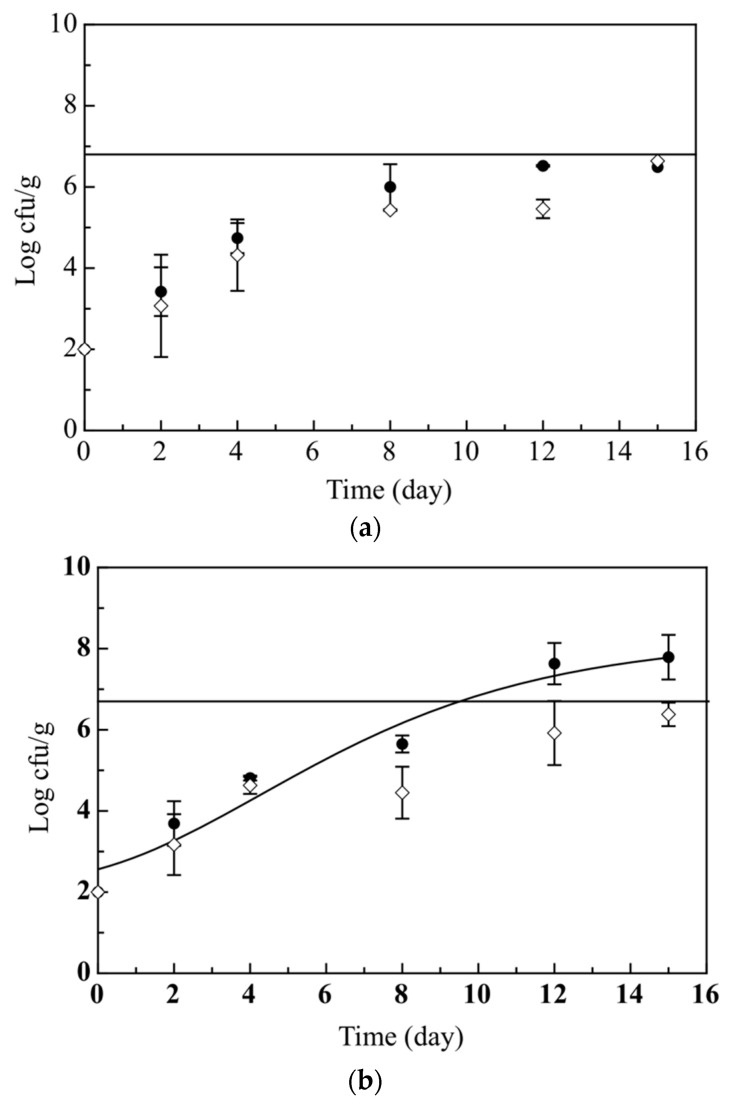
Evolution during 15 days of storage at 4 °C of total mesophilic (**a**) and psychrotrophic (**b**) bacteria in the breaded cod sticks. The curves are the best fitting of the experimental data. (●) Control breaded cod sticks without olive paste (Ctrl) (◊) Active breaded cod sticks with olive paste (Active).

In general, the quality of raw fish depends on microbial and sensory quality. The microbial quality decay of the developed breaded cod sticks (Ctrl and Active) was determined by monitoring the viable cell concentration of the total mesophilic and psychrotrophic bacteria (TMB, TPB), *Pseudomonas* spp. (Pse), *Shewanella* (Shew.) and *Photobacterium* (Phot.). The evolution of total mesophilic and psychrotrophic bacteria was reported in Figure 1a,b. As can be seen, the microbial cell concentration in both Ctrl and Active samples gradually increased with time. In particular, the cell concentration of total mesophilic bacteria is very similar between Ctrl and Active samples and both of them did not exceed the microbial limit set at 5 × 10^6^ cfu/g. In contrast, a significant difference was observed for psychrotrophic bacteria, since the cell concentration in the Ctrl samples on the 15th day of monitoring was higher compared to the Active samples. In fact, the Ctrl exceeded the microbial limit (5 × 10^6^ cfu/g) after 9.05 days, while the Active samples maintained the microbial concentration below the limit until the 15th day of storage.

It is well known that the deterioration of fish products could be characterized by the presence of H_2_S off-odors and off-flavors, caused by specific microbial groups [5]. For this reason, the microbial concentration of *Pseudomonas* spp., hydrogen-sulfide-producing bacteria (HSPB-*Shewanella*), and psychrotolerant and heat-labile aerobic bacteria (PHAB-*Photobacterium phosphoreum*) were also monitored.

**Figure 2:**
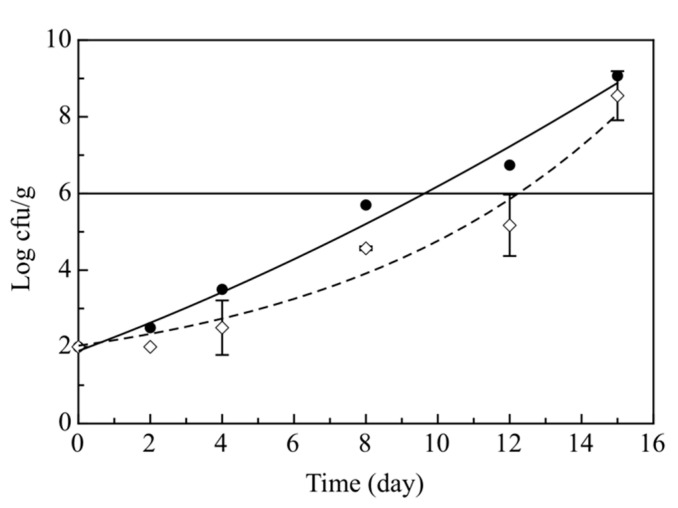
Evolution during 15 days of storage at 4 °C of *Pseudomonas* spp. in the breaded cod sticks. The curves are the best fitting of the experimental data. (●) Control breaded cod sticks without olive paste (Ctrl) (◊) Active breaded cod sticks with olive paste (Active).

Figure 2 shows the evolution of *Pseudomonas* spp. as a function of storage days. A substantial difference between the Ctrl and the Active samples was also found. Although both samples exceeded the microbial limit set at 10^6^ cfu/g, the microbial concentration of Active samples, for almost the entire monitoring time, remained below the control by about one log cycle. As can be seen in Figure 2, *Pseudomonas* spp. growth was slower in the Active samples compared to the control, thus suggesting that the bioactive compounds present in dry olive paste acted as antimicrobial agents [34]. Therefore, the phenolic compounds used to enrich the breading of cod sticks exerted an inhibitory effect against *Pseudomonas* spp. [40] and this could justify the two different trends of data recorded for sticks with and without olive paste.

**Figure 3:**
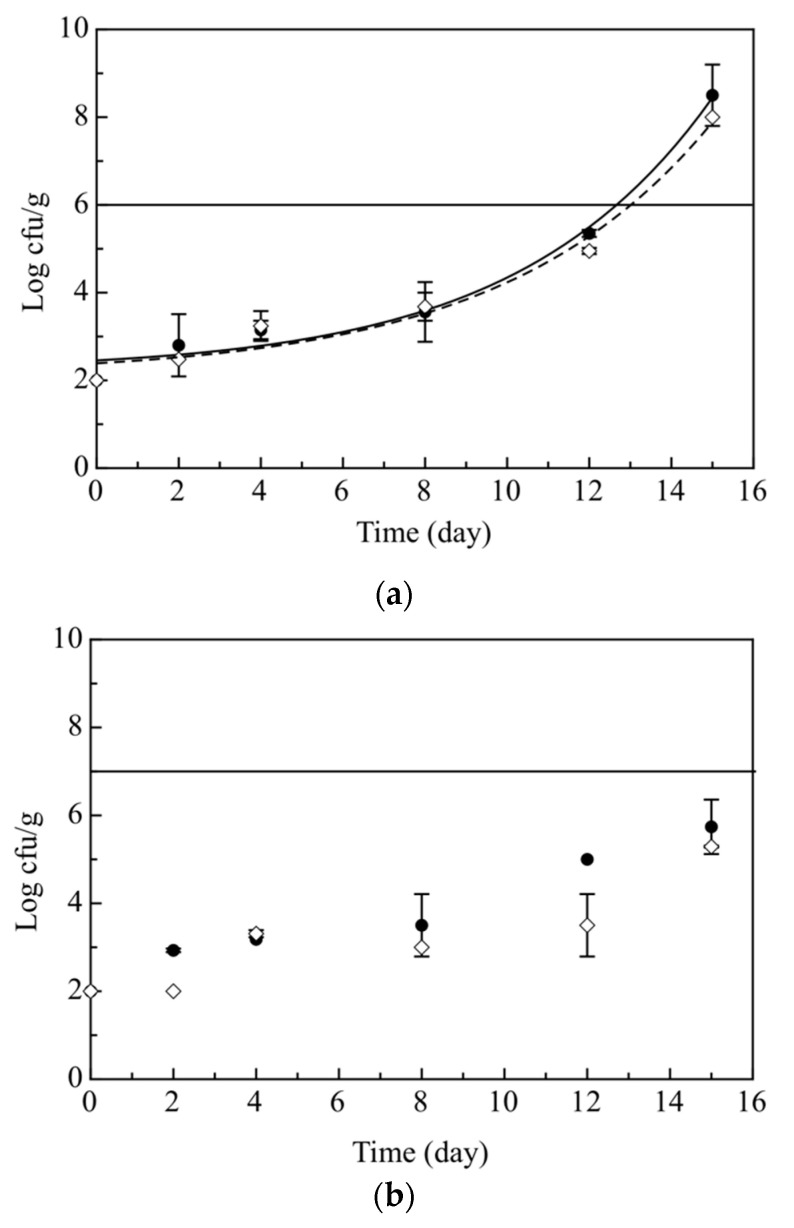
Evolution during 15 days of storage at 4 °C of *Shewanella* (**a**) and *Photobactherium* (**b**) in the breaded cod sticks. The curves are the best fitting of the experimental data. (●) Control breaded cod sticks without olive paste (Ctrl) (◊) Active breaded cod sticks with olive paste (Active).

As regards the behavior of *Shewahella* and *Photobacterium*, the microbial growth was very similar in both Ctrl and Active samples (Figure 3a,b). In both cases, a gradual increase in microbial concentration was observed during the 15 days of storage. Specifically, for *Shewanella* (Figure 3a) the Active samples did not differ substantially from the control, as both of them exceeded the microbiological acceptability limit (10^6^ cfu/g) almost on the same day of observation (13.01 and 12.66, respectively). On the other hand, the cell concentration of *Photobacterium* in Ctrl and Active samples was below the microbiological acceptability limit (10^7^ cfu/g) for the entire storage time.

Based on the results obtained by the microbiological analyses, the fitting of experimental data allowed calculating the microbiological acceptability limit (MAL). All the MAL values were summarized in Table 2 and comparing data recorded for specific spoilage groups, total mesophilic bacteria, and *Photobacterium* may be considered of less importance for the evaluation of the microbial quality of this fish product, with their cell concentrations lower than those required to spoil raw fish [30]. For the other investigated microbial groups, LAB and Enterobacteriaceae, a general inhibition was found in the Active samples. The presence of dry olive paste in the breading exerted an antimicrobial activity, slowing down and keeping the cellular loads lower than those of the control (data not shown). Several studies show that some phenols and flavonoids (hydroxytyrosol, tyrosol, and luteolin) of olive oil by-products have an antimicrobial effect especially against Gram-positive bacteria [22,41]. Our results also showed that phenolic compounds of olive paste affected Gram-negative bacteria such as *Pseudomonas* ssp., Enterobacteriaceae and *Photobacterium*.

During the 15 days of storage, in the Ctrl the pH remained roughly between values of 7.12 and 6.96, while in the Active samples between 7.0 and 6.40 (data not shown). This slight difference in pH between Ctrl and Active samples could not be attributed to relevant differences in microbial proliferation, but it is most probably ascribed to the olive paste itself which is slightly acidic [42,43].

The antimicrobial effect exerted by olive by-products has also been well evaluated by Kuley et al. [40]. These authors observed the antimicrobial effect of by-products against various food-borne pathogens and fish spoilage bacteria, thus promoting their use as valid food additives. Similar results were also reported by other researchers with other natural extracts. In particular, Martínez et al. [20] observed that the extract obtained from olives, pomegranate, and rosemary reduced microbial growth in fish patties. Danza et al. [28] observed that bio-citrus (500 ppm) in fish burgers exerted an antimicrobial effect against *Pseudomonas* spp., HSPB, and PHAB, keeping their growth lower than the control up to 13 days of storage. On the other hand, Del Nobile et al. [31] evaluated the combined effects of MAP and three natural extracts (thymol, lemon, and grapefruit seed) on fresh blue fish burgers. They observed that the combined effect of MAP and natural extracts maintained the microbial quality of products, exerting an antimicrobial effect against the main spoilage microorganisms of fish.

### 3.3. Sensory Quality of Breaded Cod Sticks

The expert panel judged the general appearance, the color, the odor, the texture, and the overall quality of breaded cod sticks. Scores recorded from Ctrl and Active samples for the four specific sensory attributes are reported in Table 3, as mean with relative standard deviation. As can be inferred from data in the table, for eight days of storage, both types of cod sticks, with and without olive paste, were perceived fully acceptable. After this storage time, some differences start to appear between Ctrl and Active fish, above all in terms of the odor parameter, generally considered the most critical sensory attribute for fish products [13]. As a fact, Ctrl products after 12 days were considered no more acceptable fue to unpleasant odor, whereas cod sticks with active breading remained acceptable for one more day. The other sensory attributes maintained a better trend in the Active samples than in the Ctrl fish.

**Figure 4:**
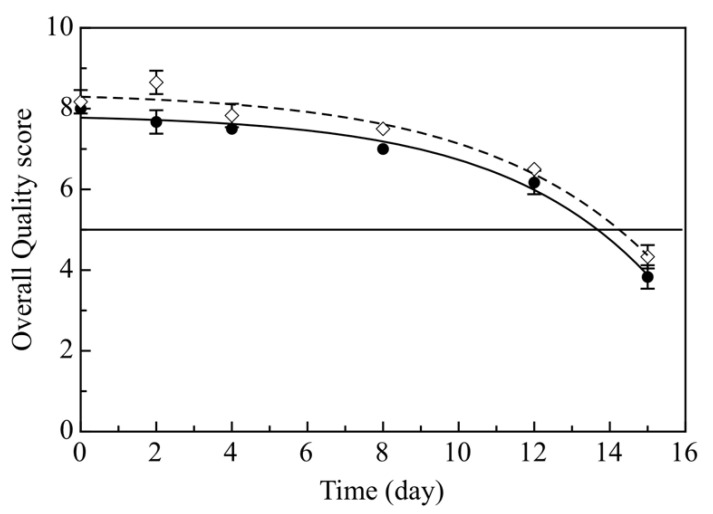
Overall Quality evolution during 15 days of storage of breaded cod sticks. The curves are the best fitting of the experimental data. (●) Control breaded cod sticks without olive paste (Ctrl) (◊) Active breaded cod sticks with olive paste (Active).

In Figure 4 data with fitting curves of global sensory quality were reported. As can be seen, both Ctrl and Active samples were positively judged by the panel, in fact for most of the monitoring days the samples obtained a score between 8 and 6, corresponding to a good quality product. As expected, a gradual decrease was observed, more emphasized after the first week of storage. It is worth noting that the Ctrl samples maintained the Overall Quality score slightly lower than the Active one, and so the samples without any olive paste became unacceptable before the Active fish products. Specifically, the Ctrl exceeded the limit (score = 5) after 13.67 days of storage, while the Active samples exceeded the limit after 14.24 days (SAL values reported in Table 2). These results show that adding the olive paste to breading does not worsen the quality of cod sticks, on the contrary, it contributes to retaining sensory quality. Another striking future of sensory data recorded for cod sticks is that the panelists did not consider negatively the slightly darker color of the breading. On the contrary, Cedola et al. [20] reported that the presence of this olive by-product in fish burgers initially compromised the overall quality, due to worsening of color and texture. Our finding suggests that the application of the olive paste to the breading of cod sticks is very appropriate.

### 3.4. Breaded Cod Sticks Shelf Life

To determine final product shelf life, both fitting values of microbiological (MAL) and sensory quality (SAL) were taken into account. To this aim, data collected in Table 2 can be considered. In particular, the shelf life of breaded cod sticks was reported in the last column of this table as the lowest value between the MAL referred to all spoilage bacteria and the SAL. As a fact, the factor that influenced the breaded cod sticks’ shelf life more significantly was the microbial proliferation. In particular, psychrotrophic bacteria, *Pseudomonas* spp. and *Shewanella* affected the shelf life of the Ctrl samples, while the growth of *Pseudomonas* spp. and *Shewanella* influenced the shelf life of the Active cod sticks. These results highlighted that the shelf life of Active samples (12.23 days) was longer than the Ctrl, for which a shelf life of 9.05 days was recorded. Similar results have been reported by Martínez et al. [19], who also applied bioactive compounds from food matrixes of vegetable origin on fish. In particular, these authors observed that the extract obtained from olives, pomegranate, and rosemary acted as a preservative in fish patties, extending the shelf life for 11 days. Danza et al. [28] also noted that dipping whole fish fillets in bio-citrus extract prior to mincing and forming fish burgers proved to be the best preserving strategy for its shelf life.

Based on our findings, breading enriched with olive paste is a useful approach to valorize an industrial by-product, enhance the nutritional properties of cod sticks, and extend their shelf life because the bioactive compounds entailed better stability of the product from both the microbiological and sensory point of view [39,40]. It is also worth considering that the current study was carried out in refrigerated conditions and that proper packaging of cod samples under MAP [12,13] could promote synergic effects with active breading, thus giving further more significant shelf-life prolongation.

## 4. Conclusions

In this study, dried olive paste, as a valuable by-product of the modern olive oil production process, was applied as breading to cod sticks to prolong their shelf life. To this aim, the microbiological, sensory, and chemical qualities of the breaded cod sticks were assessed during two weeks of refrigerated storage. The results show that the olive paste used in the breading increased the phenol and flavonoid content of cod sticks and, as a consequence, its antioxidant activity. In addition, the fortification maintained the microbiological quality of the active samples better than the control by more than three days and the sensory acceptability of the product was not compromised. In fact, an extension of the shelf life of active samples was observed. While the control cod sticks remained acceptable for about nine days, the active fish products were acceptable up to more than 12 days. Therefore, this was a concrete example of sustainable reuse of olive oil by-products to develop a breaded cod stick with healthful properties.

## Figures and Tables

**Table 1:** Total phenols, total flavonoids, and antioxidant activity of raw and cooked sticks.

Samples	Total Phenols (mg GAE/g dw) ± SD	Total Flavonoids (mg QE/g dw) ± SD	Antioxidant Activity (mg Trolox/g dw) ± SD
R-Ctrl	2.70 ± 0.15 ^a,A^	1.69 ± 0.09 ^a,A^	5.88 ± 0.18 ^a,A^
R-Active	12.63 ± 0.18 ^b,A^	13.68 ± 0.90 ^b,A^	20.02 ± 0.43 ^b,A^
C-Ctrl	2.82 ± 0.13 ^a,A^	1.38 ± 0.13 ^a,B^	4.40 ± 0.15 ^a,B^
C-Active	12.46 ± 0.26 ^b,A^	10.61 ± 0.53 ^b,B^	12.55 ± 0.75 ^b,B^

Data in columns with different superscript lowercase letters are significantly different (R-Ctrl and R-Active; C-Ctrl and C-Active), while with different superscript uppercase letters they are different between raw and cooked (R-Ctrl and C-Ctrl; R-Active and C-Active) (*p* < 0.05); GAE: gallic acid equivalent; QE: quercitin equivalent; R-Ctrl: raw breaded Cod sticks without olive paste; C-Ctrl: cooked breaded Cod sticks without olive paste; R-Active: raw breaded Cod sticks with olive paste; C-Active: cooked breaded Cod sticks with olive paste.

**Table 2:** Shelf life (day) of breaded cod sticks (Ctrl) and Active breaded cod sticks (Active) as the lowest value among microbiological acceptability limit vales, referred to each spoilage group (MAL for total psychrotrophic and mesophilic bacteria—TPB and TMB, *Pseudomonas* spp.—Pse., *Shewanella*—Shew. and *Photobacterium*—Phot.) and sensory acceptability limit (SAL for overall quality).

Samples	Microbiological Acceptability Limit (Day)					Sensory Acceptability Limit (Day)	Shelf Life (Day)
	MAL^TPB^	MAL^TMB^	MAL^Pse.^	MAL^Shew.^	MAL^Phot.^	SAL	
Ctrl	9.05 ± 0.87 ^a^	>15	9.62 ± 0.55 ^a^	12.66 ± 0.57 ^a^	>15	13.67 ± 0.38 ^a^	9.05 ± 0.87 ^a^
Active	>15	>15	12.23 ± 0.72 ^b^	13.01 ± 0.68 ^a^	>15	14.24 ± 0.45 ^a^	12.23 ± 0.72 ^b^

Data (±SD *n* = 2) with different superscript letters in each column are significantly different (*p* < 0.05). Ctrl: breaded cod sticks without olive paste; Active: breaded cod sticks with olive paste.

**Table 3:** Sensory quality of breaded cod sticks in terms of appearance, odor, color, and texture.

Sensory Attributes	Samples	Storage Time (Day)					
		0	2	4	8	12	15
Appearance	Ctrl Active	8.0 ± 0.0 ^a^ 8.0 ± 0.0 ^a^	8.0 ± 0.0 ^a^ 8.0 ± 0.0 ^a^	8.0 ± 0.0 ^a^ 8.0 ± 0.0 ^a^	7.50 ± 0.0 ^a^ 7.50 ± 0.0 ^a^	6.17 ± 0.29 ^a^ 7.0 ± 0.0 ^b^	4.33 ± 0.29 ^a^ 6.17 ± 0.29 ^b^
Color	Ctrl Active	8.0 ± 0.0 ^a^ 8.0 ± 0.0 ^a^	8.0 ± 0.0 ^a^ 8.0 ± 0.0 ^a^	8.0 ± 0.0 ^a^ 8.0 ± 0.0 ^a^	7.33 ± 0.29 ^a^ 7.33 ± 0.29 ^a^	6.33 ± 0.29 ^a^ 7.0 ± 0.0 ^b^	4.83 ± 0.29 ^a^ 5.17 ± 0.29 ^a^
Odor	Ctrl Active	8.17 ± 0.29 ^a^ 8.50 ± 0.0 ^a^	7.50 ± 0.0 ^a^ 8.33 ± 0.29 ^b^	7.33 ± 0.29 ^a^ 7.83 ± 0.29 ^a^	7.0 ± 0.50 ^a^ 7.0 ± 0.50 ^a^	5.33 ± 0.29 ^a^ 6.17 ± 0.29 ^b^	3.67 ± 0.29 ^a^ 4.50 ± 0.0 ^b^
Texture	Ctrl Active	8.50 ± 0.0 ^a^ 8.50 ± 0.0 ^a^	8.33 ± 0.29 ^a^ 8.33 ± 0.29 ^a^	8.0 ± 0.0 ^a^ 8.0 ± 0.0 ^a^	7.83 ± 0.29 ^a^ 8.00 ± 0.0 ^a^	6.33 ± 0.29 ^a^ 6.83 ± 0.29 ^a^	5.0 ± 0.0 ^a^ 6.0 ± 0.50 ^b^

^a,b^ Data (±SD *n* = 2) with different superscript letters in each column for each attribute are significantly different (*p* < 0.05). Ctrl = breaded cod sticks without olive paste; Active = breaded cod sticks with olive paste.
